# Cavernous Hemangioma of the Conjunctiva

**DOI:** 10.18502/jovr.v16i2.9097

**Published:** 2021-04-29

**Authors:** Noopur Deokinandan Nayak Shinkre, Ugam P.S. Usgaonkar

**Affiliations:** ^1^Department of Ophthalmology, Goa Medical College, Goa, India

##  PRESENTATION

A 23-year-old healthy male presented to our outpatient department with complaint of foreign body sensation and a painless, red mass on the nasal side of his right eye, which he had noticed approximately six months before. It had gradually increased to attain its present size.

**Figure 1:**
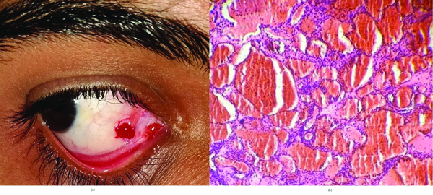
(a) Image showing a bright red, smooth, lobulated mass on the nasal side of the bulbar conjunctiva with tortuous conjunctival vessels at its base. (b) Histopathological evaluation of the lesion showing multiple, blood-filled cavernous spaces surrounded by a fibromyxoid stroma (hematoxylin and eosin, 10× magnification).

On examination, his visual acuity on Snellen's chart was 20/20 in both eyes. Examination of his right eye revealed a bright red, vascular, smooth, lobulated mass on the nasal side of the bulbar conjunctiva, measuring approximately 5×5×4 mm, with tortuous and engorged conjunctival vessels at the base of the lesion [Figure 1a]. The lesion was mobile and did not exhibit pulsations. Other findings of his ocular examination were unremarkable. No similar lesions were detected elsewhere on his body.

**Figure 2:**
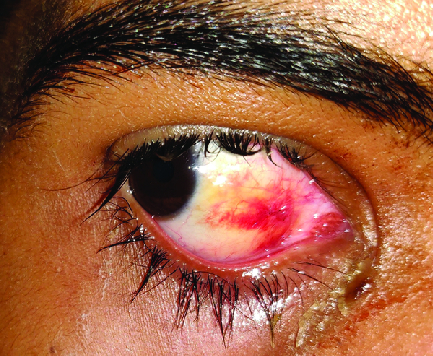
Image acquired one day after excision of the lesion. The conjunctival defect is apposed with 10–0 nylon sutures.

A clinical diagnosis of conjunctival hemangioma was made. The lesion was surgically excised under topical anesthesia. No extension was noted beyond the tenon's capsule. Histopathological examination of the lesion revealed a cavernous hemangioma of the bulbar conjunctiva [Figure 1b]. Postoperatively, the patient was treated with topical antibiotics and steroid eye drops [Figure 2].

##  DISCUSSION

Cavernous hemangiomas are vascular malformations characterized pathologically by large, thin-walled, and cystically dilated blood vessels. They are rare vascular tumors of the ocular surface; unlike capillary hemangioma, lymphangioma, and pyogenic granuloma which are more common.^[1,2]^

Occurrence of an isolated cavernous hemangioma of the conjunctiva has rarely been reported to date. In addition to the results of the surveys conducted by Elsas et al^[3]^ and Shields et al,^[2,4]^ to the best of our knowledge, only nine cases have been reported in the English literature, and notably, most of them have occurred in young males. The reported lesions arose from either the caruncle or the temporal side of the bulbar conjunctiva. However, in our case, unlike the previously reported cases, the tumor was located on the nasal side of the bulbar conjunctiva, neither overlying nor involving the caruncle, thus making it, to the best of our knowledge, the first such case to be reported.

In these cases, the primary concern is purely cosmesis. However, some cases may be associated with recurrent subconjunctival hemorrhage. Excisional biopsy of the tumor is considered to be the treatment of choice.^[5]^

##  Declaration of Patient Consent

The authors certify that they have obtained all appropriate patient consent forms. In the form the patient has given his consent for his images and other clinical information to be reported in the journal. The patient understand that his name and initial will not be published and due efforts will be made to conceal his identity, but anonymity cannot be guaranteed.

##  Financial support and sponsorship

Nil.

##  Conflicts of interest

There are no conflicts of interest.
